# Integrating Molecular Semantics and Three-Dimensional Geometry for Critical Property Prediction: A Multimodal GNN-BERT Framework

**DOI:** 10.3390/molecules31183251

**Published:** 2026-09-14

**Authors:** Beibei Wang, Zhuoyao Lv, Nan Ning, Yichen Zhang, Jiquan Zhang

**Affiliations:** 1 School of Emergency Management of Jilin Province, Changchun Institute of Technology, No. 3066 Tongzhi Street, Changchun 130021, China; 2 School of Environment, Northeast Normal University, Changchun 130117, China

**Keywords:** critical properties, 3D molecular information, multimodal learning, SMILES, GNN-BERT

## Abstract

Accurate prediction of critical temperature, pressure, and volume is essential for thermodynamic modeling and process safety design, yet remains challenging for complex molecules under data-scarce conditions. Here, we develop a multimodal GNN-BERT framework that integrates SMILES-based chemical semantics with two-dimensional topology and three-dimensional molecular geometry for critical property prediction. BERT captures molecular sequence information, while graph neural networks learn topology- and geometry-aware representations through message passing. Evaluation on 913 chemical compounds demonstrates that the proposed framework consistently outperforms conventional machine-learning models and single-modality baselines. Importantly, comparative analyses among BERT, BERT+2D-GNN, and BERT+3D-GNN reveal that incorporating three-dimensional molecular geometry provides a consistent 5–10% improvement across critical temperature, pressure, and volume prediction. Additional validation using random forest and support vector regression further confirms that the predictive contribution of 3D molecular information is not architecture-dependent. These results highlight three-dimensional molecular geometry as an important structural parameter for data-driven critical property prediction and provide a reliable computational strategy for thermodynamic estimation and chemical process safety applications.

## 1. Introduction

With the rapid development of the chemical industry, countless chemicals have rapidly entered human production and daily life. When chemicals are in a gas–liquid critical state and conditions or environments change, their phases may undergo rapid transformations, potentially leading to explosions. Therefore, strengthening the management and prevention of hazardous chemicals is crucial. Fully understanding the properties of hazardous chemicals enables the identification and assessment of potential risks at their source [1]. Critical properties (including critical temperature, critical pressure and critical volume) are of fundamental significance in materials science, chemical engineering, safety and disaster prevention and mitigation [2,3,4]. They define the limit boundary within which a substance can exist in a stable liquid state [5]. Once operating conditions exceed this boundary, violent and fundamental state changes occur, typically accompanied by massive energy release [6], leading to explosions, leaks, and accidents [7]. More precise knowledge of these boundary values facilitates the provision of technical recommendations for hazardous incidents such as heat generation, thermal runaway, explosions, and leaks from a chemical reaction engineering perspective [8].

Traditionally, experimental determination has been the primary method for obtaining critical properties of organic compounds, such as pulse heating and capillary methods [9]. However, this approach has numerous limitations. For macromolecular compounds prone to decomposition at high temperatures, experimental determination is not only challenging but may also yield inaccurate results. Currently, predictive studies of compound critical properties primarily rely on two major categories of computational methods. The first category comprises equations of state (Peng–Robinson, Soave–Redlich–Kwong) and their modified forms [10]. These approaches correlate temperature, pressure, and volume by fitting limited experimental data, offering practical utility for extrapolating properties of homologues with few known values. The second category encompasses data-driven methods based on quantitative structure–property relationships [11,12,13]. Conventional quantitative structure–property relationship (QSPR) approaches employ handcrafted molecular descriptors [14], including structural fingerprints and topological indices, in conjunction with deep learning methodologies to construct statistical correlations between molecular structure and physicochemical properties [15,16]. In early research, Espinosa et al. [17] employed QSPR based on fuzzy ARTMAP to predict Tc for 530 compounds. The work of Zhou et al. [18] and Yao et al. [19] demonstrated the feasibility of linear methods like multiple linear regression (MLR). Joback and Reid [20] in 1987 proposed a foundational group-contribution method for estimating pure-component properties, which linearly sums predefined functional-group contributions and remains one of the most widely adopted classical approaches for critical-property prediction. Subsequently, Wang et al. [21] applied nonlinear methods such as Support Vector Machines (SVM) and Radial Basis Function Neural Networks (RBFNN) to prediction tasks. In recent years, more advanced algorithms like Multi-Layer Perceptron Artificial Neural Networks (MLP-ANN) have been introduced, exemplified by the research of Beghour and Lahiouel [22].

However, these approaches still face several challenges. Equation-of-state methods generally require experimentally derived component-specific parameters, which limits their applicability to newly synthesized compounds or molecules lacking sufficient experimental measurements. Moreover, traditional QSPR approaches based on molecular descriptors have demonstrated considerable success in establishing structure–property relationships by capturing important chemical and topological characteristics. Nevertheless, the predictive capability of descriptor-based models is often constrained by the selection and design of predefined descriptors. In particular, conventional descriptors primarily characterize molecular composition, functional groups, and two-dimensional connectivity, while the three-dimensional spatial arrangement and molecular geometry associated with intermolecular interactions are often insufficiently represented. This limitation may result in incomplete molecular representations and restrict the transferability of QSPR models for complex critical property prediction. To address this challenge, this study integrates simplified molecular input line entry system (SMILES) sequence information with two-dimensional molecular topology and three-dimensional molecular geometry, aiming to construct a more comprehensive molecular representation for critical property prediction.

Breakthroughs in artificial intelligence technology, particularly the successful application of deep learning in natural language processing (NLP) and graph learning, have provided novel solutions to the aforementioned challenges. Compounds are essentially topological structures formed by atoms connected through chemical bonds, making them naturally amenable to representation as graph data. Graph Neural Networks (GNNs) [23], as powerful tools specifically designed to process non-Euclidean data, can effectively learn both local and global features of atoms (nodes) and chemical bonds (edges) through message-passing mechanisms. This capability demonstrates significant potential in molecular property prediction tasks. Meanwhile, pre-trained language models exemplified by BERT (Bidirectional Encoder Representations from Transformers) [24] have achieved revolutionary breakthroughs. Through self-supervised pre-training tasks like masked language modeling on large-scale unlabeled corpora, BERT can deeply understand the contextual semantics of language. Notably, SMILES [25] strings for molecules serve as a highly structured “chemical language,” whose inherent syntactic rules and semantic information can similarly be mined by analogous techniques. At the molecular representation level, researchers have primarily explored three technical approaches. The first approach uses linear molecular representations (such as SMILES strings) as input and leverages natural language processing techniques to learn molecular semantics. ChemBERTa, proposed by Chithrananda et al. [26], demonstrated through large-scale self-supervised pretraining that chemical language models can effectively encode molecular structural information. Ross et al. [27] further confirmed that deep SMILES-based representations can capture subtle correlations between molecular properties, laying the theoretical foundation for sequence-driven property prediction. The second approach focuses on two-dimensional molecular graph structures, utilizing graph neural networks (GNNs) to propagate messages between atoms and chemical bonds in order to learn topological features. Yang et al. [28] systematically analyzed the behavior of message-passing networks in molecular representation learning, while Gasteiger et al.’s [29] Directed Message-Passing framework (DimeNet) enhanced geometric awareness by incorporating bond angle information, significantly improving the prediction accuracy of quantum mechanical properties. The third approach takes 3D atomic coordinates directly as input and models spatial configurations using isomorphic or invariant neural networks. SchNet was the first to introduce continuous filtering convolution into molecular modeling, enabling efficient learning of quantum interactions; the Uni-Mol framework proposed by Zhou et al. [30]. established a general paradigm for 3D molecular representation learning, demonstrating the immense potential of monomodal 3D information in molecular understanding. Recent studies demonstrate that SMILES-based pre-trained models can acquire profound chemical knowledge, offering valuable complementary perspectives for molecular characterization [31]. In recent years, applying SMILES to BERT models for predicting molecular properties has yielded significant results in regression and classification tasks. Successful approaches include Zheng et al.’s [32] SMILES sequence pre-training model based on BERT, Barranco-Altirriba et al.’s [33] Smile-to-BERT model, and Mswahili et al.’s [34] exploration of positional encoding for SMILES. These methods have also been applied to predicting explosion consequences, as demonstrated in Wang et al.’s [35] research. In addition to the studies mentioned above, there are many other studies [36] that have utilized the BERT model.

Although the aforementioned single-modal methods have achieved significant progress in their respective application scenarios, molecules are inherently complex objects with multiple representations: SMILES sequences embody chemical syntax and functional group context; two-dimensional graph structures explicitly express atomic connectivity and electron delocalization characteristics; and three-dimensional conformations carry geometric information regarding steric hindrance, stereochemistry, and intermolecular interactions. Any single perspective inevitably leaves information gaps. Consequently, the fusion of multi-source molecular representations has become a cutting-edge trend. The Molecular Attention Transformer (MAT), proposed by Maziarka et al. [37], attempts to combine distance matrices with SMILES embeddings; Luo et al. [38], meanwhile, explored the feasibility of a single Transformer architecture simultaneously understanding two-dimensional topology and three-dimensional geometry. However, in the field of engineering thermodynamics—particularly in the task of predicting critical properties—how to effectively couple sequence semantics, two-dimensional topology, and three-dimensional geometric information remains an under-explored topic. Existing research has largely focused on the prediction of quantum mechanical properties or biological activity; multimodal deep learning methods targeting macroscopic thermodynamic properties—particularly critical pressure, which is highly sensitive to intermolecular interactions and exhibits significant heterogeneity in experimental data—remain scarce.

Based on this, this study innovatively proposes a hybrid deep learning model integrating GNN and BERT (GNN-BERT), aiming to synergistically leverage both the three-dimensional topological structure information of compounds and the deep semantic information of two-dimensional sequences to achieve more precise and robust predictions of their critical properties. Specifically, the model captures spatial connectivity features of molecular graphs through a GNN branch, while simultaneously decoding SMILES sequences via a BERT branch. These two modalities’ features are then effectively integrated to jointly drive downstream regression prediction tasks. This dual-channel fusion strategy is expected to overcome the information bottlenecks of single-model approaches, enabling more comprehensive characterization of compound structure–property relationships. This offers a powerful computational tool for rapid screening and performance evaluation of complex compounds. This research not only explores a novel pathway for predicting critical properties but also provides valuable insights for applying multimodal information fusion in chemoinformatics.

## 2. Methodology

### 2.1. Dataset

**Figure 1:**
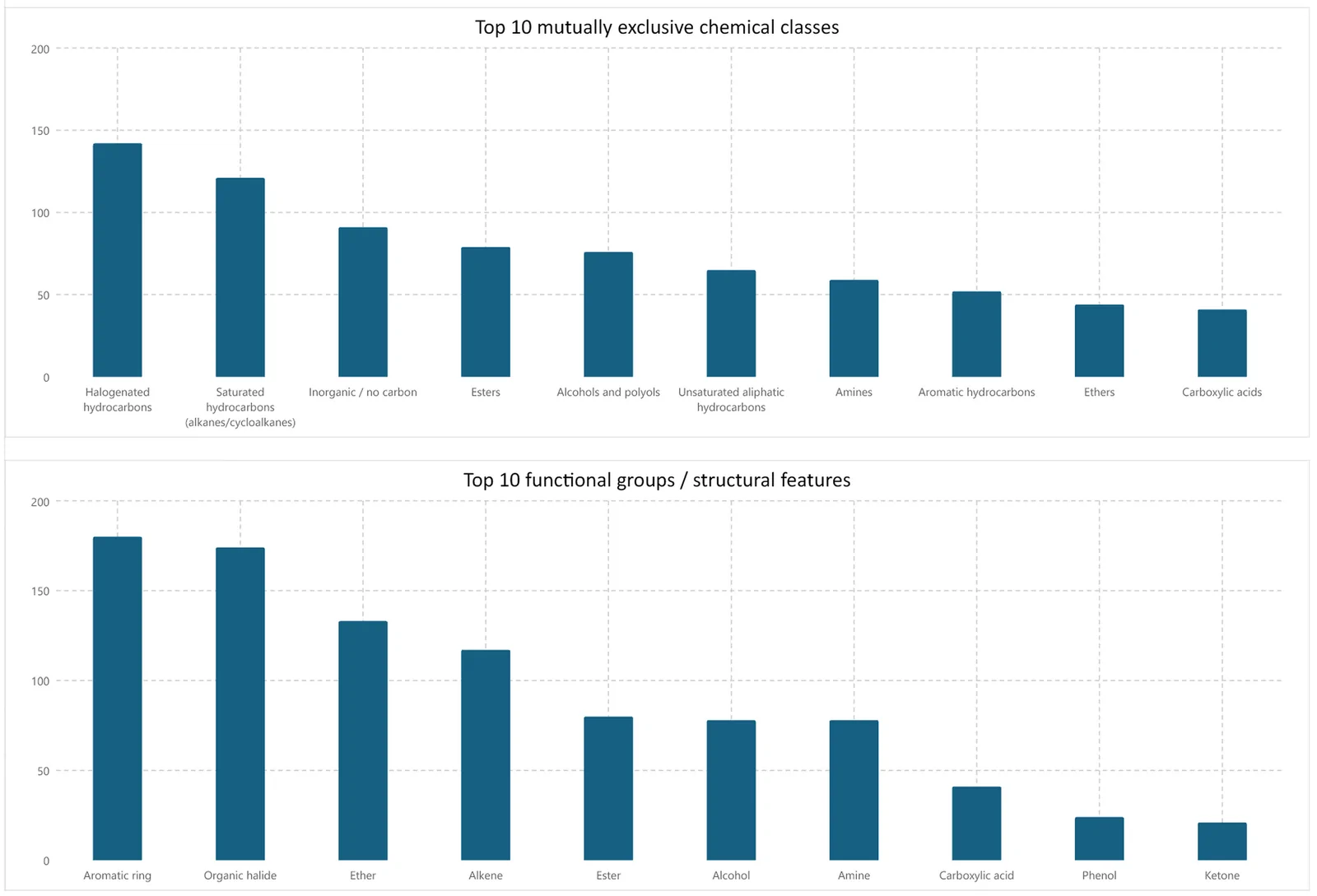
Types of Compounds Included in the Dataset.

This dataset comprises critical thermodynamic properties for 913 compounds collected from the literature [21,39], covering a broad range of chemical classes, including hydrocarbons, aldehydes, alcohols, carboxylic acids, amines, benzene derivatives, sulfur-containing compounds, and other organic compounds. The dataset contains 911 critical-temperature records, 596 critical-pressure records, and 383 critical-volume records. All compounds were represented using their canonical SMILES strings. The chemical diversity of the dataset was quantified using SMILES-based structural classification. All 913 unique SMILES were successfully parsed. Under a mutually exclusive main-class scheme, no single chemical class dominated the dataset. Halogenated hydrocarbons constituted the largest class (142 compounds, 15.6%), followed by saturated hydrocarbons, including alkanes and cycloalkanes (121, 13.3%), inorganic or carbon-free compounds (91, 10.0%), esters (79, 8.7%), alcohols and polyols (76, 8.3%), unsaturated aliphatic hydrocarbons (65, 7.1%), amines (59, 6.5%), and aromatic hydrocarbons (52, 5.7%). SMARTS-based, non-exclusive substructure analysis showed that the most frequent structural features were aromatic rings (19.7%), organically bound halogens (19.1%), ether groups (14.6%), and non-aromatic C=C bonds (12.8%). These results demonstrate that the dataset covers a relatively broad chemical space rather than being dominated by alkanes. Nevertheless, predictions for sparsely represented classes, such as amides and some specialized heteroatom-containing compounds, should be interpreted cautiously because their chemical space is less extensively sampled. Figure 1 shows the types of compounds included in the dataset.

Evaluation protocol for the proposed 3DBERT-GNN model. The proposed 3DBERT-GNN model was evaluated using five-fold cross-validation to provide a more robust estimate of predictive performance. For each fold, approximately 70% of the samples were used for training, 15% for validation, and 15% for testing. The validation subset was used for model selection and early stopping, while the corresponding test subset was kept completely unseen until the final evaluation of that fold. This cross-validation protocol was applied independently to each critical-property dataset.

Input representations and preprocessing for baseline models. Conventional machine-learning baselines were constructed using molecular representations derived exclusively from structural information. For the 2D baselines, Morgan fingerprints were generated from SMILES strings. For the 2D + 3D baselines, these fingerprints were supplemented with descriptors calculated from the corresponding molecular conformations. No critical-property value or target-derived variable was used during molecular representation generation.

For each property-specific dataset, the conventional ML baselines were evaluated using a fixed 70:15:15 train/validation/test split with random state = 42. Hyperparameter selection was performed using the training and validation subsets only, whereas the test set remained isolated until final evaluation. For SVR, feature-scaling parameters were fitted on the training subset and then applied unchanged to the validation and test subsets. Random forest models were trained without feature scaling. This procedure prevented information from the validation or test subsets from entering feature preprocessing or model fitting, thereby minimizing target leakage and enabling a consistent internal hold-out comparison among the conventional ML baselines.

### 2.2. Inputs to the Model

The proposed framework was evaluated under an incremental multimodal input design with three model configurations: BERT, BERT+2D-GNN, and BERT+3D-GNN. The BERT configuration takes tokenized SMILES strings as input and learns molecular semantic representations through self-attention. The BERT+2D-GNN configuration further incorporates the 2D molecular graph derived deterministically from each SMILES string. Specifically, the adjacency matrix is incorporated into the attention calculation as a structural bias, allowing the model to account for covalent connectivity during representation learning.

**Figure 2:**
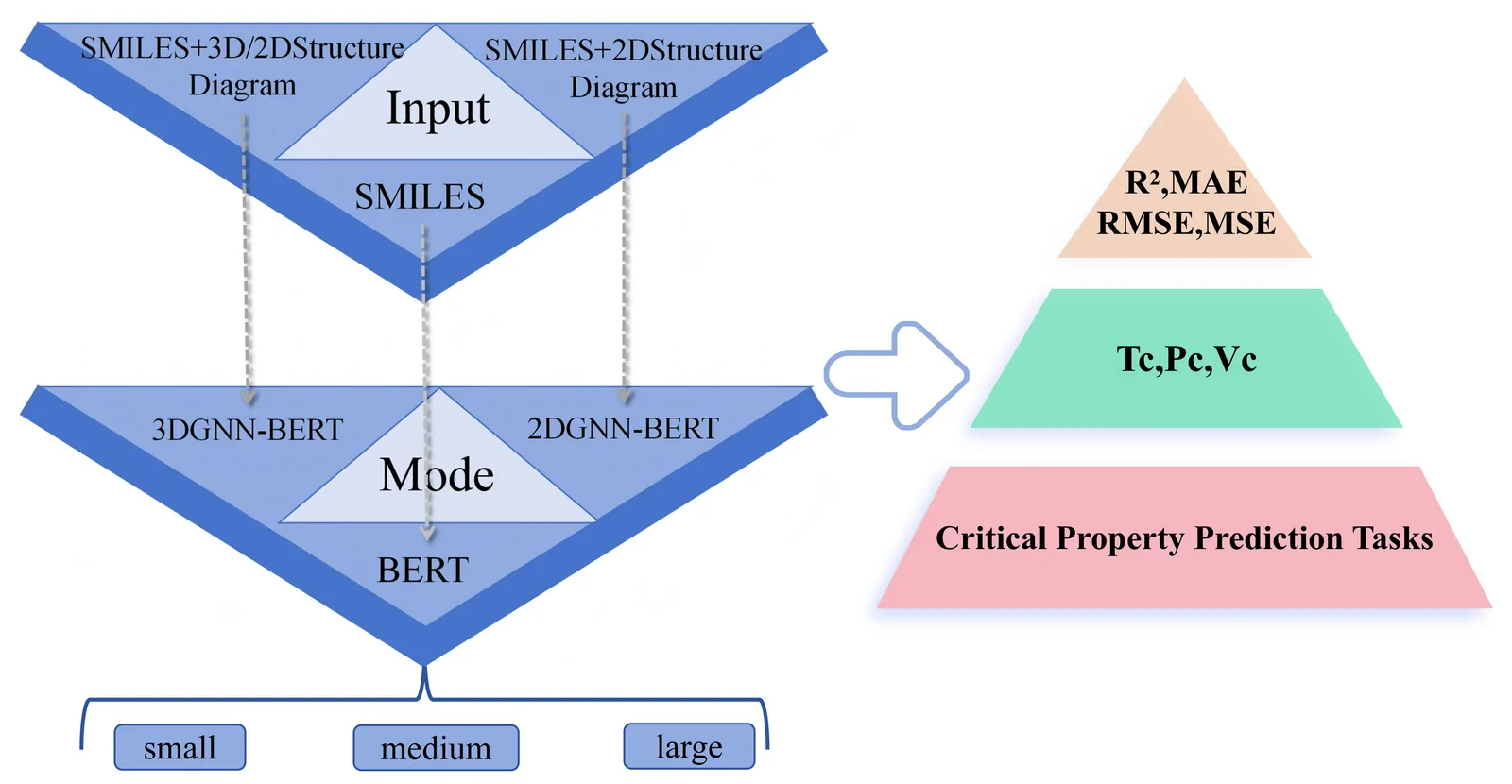
Models corresponding to three different input types.(The dashed arrows indicate the inputs to each model, while the solid arrows indicate the tasks performed by the models).

The full BERT+3D-GNN configuration integrates SMILES semantics, 2D topology, and 3D molecular coordinates. The 3D branch is implemented as an independent geometric GNN that extracts distance-based geometric features from atomic coordinates, while the 2D branch preserves explicit covalent-connectivity information. The resulting semantic, topological, and geometric representations are aligned at the atom level and integrated through learnable fusion. This incremental design enables a systematic assessment of the respective contributions of SMILES semantics, 2D topology, and 3D geometry to critical-property prediction. When 3D conformations are unavailable, the BERT and BERT+2D-GNN configurations remain applicable using SMILES-derived information alone. The three configurations are illustrated in Figure 2.

#### 2.2.1. SMILES

SMILES sequences do not directly encode topological or spatial relationships between atoms like adjacency matrices or distance matrices. Consequently, models must possess the ability to infer implicit structural information from the sequence. This process can be achieved through unsupervised learning, eliminating reliance on manually annotated data. Additionally, SMILES sequences employ specific symbols (such as ‘/’, ‘\’, ‘@’, ‘@@’) to explicitly encode stereochemical configurations. Specifically, trans and cis isomers arising from double bonds can be distinguished by the direction of the symbols “/” and “\”, while the chirality of optical isomers can be denoted by “@” and “@@”. In contrast, identifying stereoisomers from adjacency matrices and distance matrices is more complex. Adjacency matrices cannot convey stereochemical information at all. While atomic distances can reflect stereochemical differences, this requires the relative positions of at least three atoms, and stereochemical effects diminish sharply as distances increase. Therefore, training models to learn differences between stereoisomers based on distance matrices may pose greater challenges. Consequently, using SMILES sequences as model inputs demonstrates superiority over adjacency matrices and distance matrices in representing stereoisomer information.

#### 2.2.2. Molecular Geometry

2D structural diagrams illustrate the connections between atoms, including bond types (single, double, triple bonds). They clearly display functional groups within molecules, which is crucial for understanding a molecule’s reactivity and chemical properties [40].Two-dimensional molecular representations describe atomic connectivity, bond types, and functional groups, whereas three-dimensional representations provide an approximate description of molecular shape and the relative spatial arrangement of atoms. In this work, the available 3D structure files were obtained from the corresponding compound records in the PubChem database [41]. RDKit was used to read and process these files and to prepare consistent structural representations for model input. Molecular structures were processed using RDKit [42] (version 2025.09.2), an open-source cheminformatics toolkit.

The PubChem-derived 3D representation was selected because it provides a standardized, reproducible, and scalable source of coordinates for a chemically diverse dataset. Using structures obtained through a consistent workflow reduces the methodological heterogeneity that could arise if different conformer-generation or optimization procedures were applied to different compounds. The resulting representation supplies approximate spatial information complementary to the molecular connectivity encoded by SMILES. No ETKDG conformer generation, MMFF94/UFF optimization, conformer ensemble generation, or energy-based conformer selection was performed in this study.

However, the 3D coordinates used here represent only one computational conformer for each compound and should not be interpreted as an experimentally determined equilibrium liquid-phase structure. PubChem conformers are obtained from computational conformer models, and an individual conformer is not necessarily located at an energy minimum or equivalent to the lowest-energy structure in vacuum, solvent, or the liquid phase. For flexible compounds, including long-chain alkanes, multiple conformers may be thermally accessible, and their populations can vary with temperature, pressure, and the surrounding molecular environment. A single conformer therefore cannot describe the complete conformational ensemble. For polyfunctional molecules, the selected geometry may also differ from the dominant liquid-phase conformations because intramolecular and intermolecular interactions are not explicitly represented.

Accordingly, the 3D structure employed in this study is treated as an approximate auxiliary molecular representation. Conformer dependence and possible geometrical inaccuracies may introduce uncertainty into the spatial features learned by the model, especially for flexible and polyfunctional compounds. Because the model also incorporates SMILES-derived information, the prediction is not determined solely by the selected 3D conformer; nevertheless, the present approach does not explicitly account for conformational averaging. Future work could improve the representation by generating and optimizing multiple conformers and aggregating their features according to relative energies or Boltzmann populations. Molecular-dynamics sampling under conditions relevant to the measured critical properties may provide a more physically realistic description, although at substantially greater computational cost.

### 2.3. Model Architecture

GNNs are deep learning architectures specifically designed for graph data, capable of capturing the intricate relationships between nodes and edges. They are widely used in various graph analysis tasks, including vertex classification, relationship inference, and cluster recognition. The fundamental architecture of a GNN typically consists of two components: node embedding and graph convolution. Node embedding projects every vertex within a network into a compact vector space, whereas graph convolutions refine vertex attributes by integrating both embedded node representations and topological connectivity patterns, thus facilitating comprehensive representation learning across the complete network structure. In this model, the GNN incorporates spatial structural information: distance relationships between atoms, molecular spatial configurations, and interactions within three-dimensional space.

**Figure 3:**
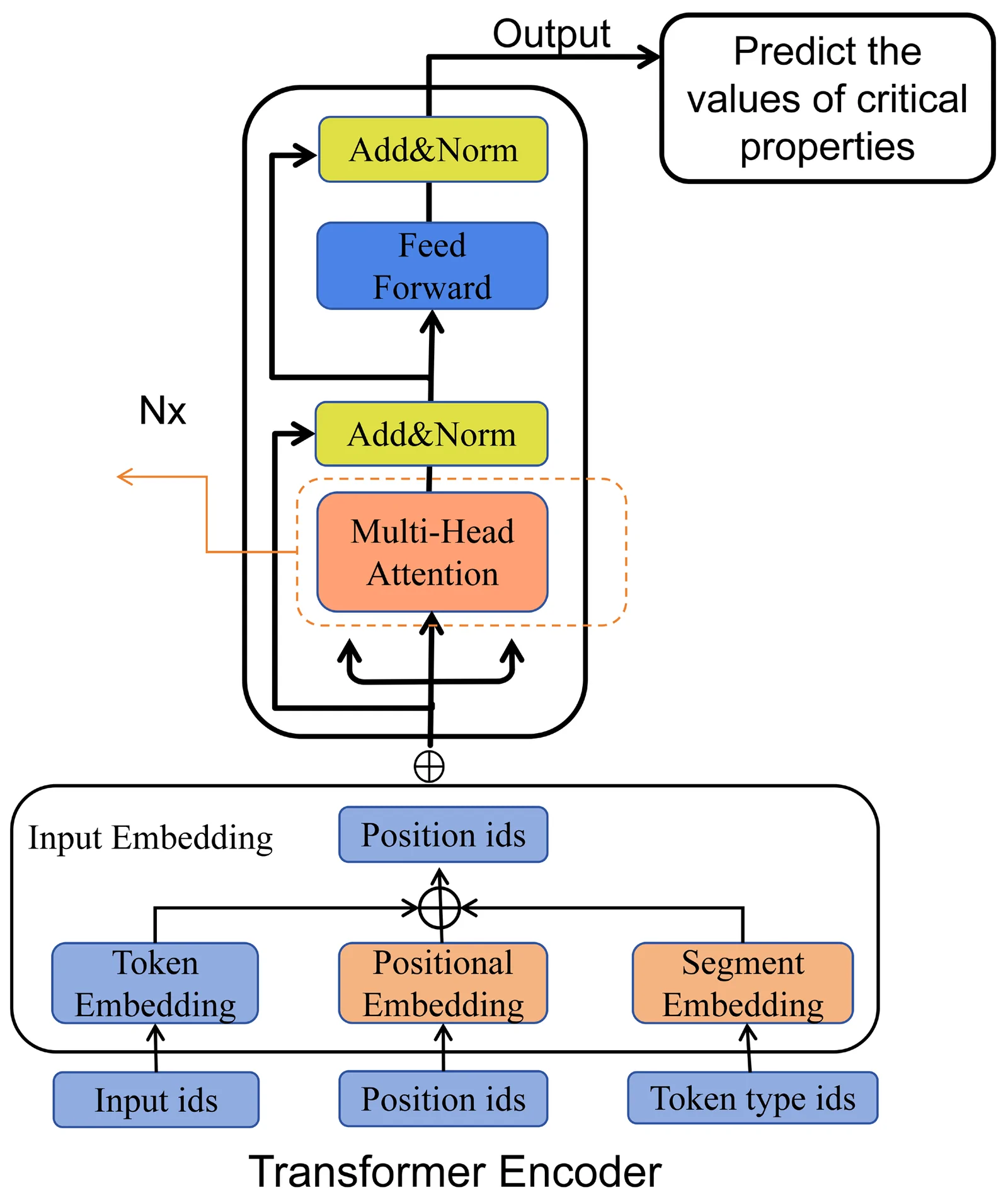
Main structure of the BERT Model.

BERT constitutes a pre-trained linguistic representation framework built upon the Transformer structure. BERT can be pre-trained using large-scale compound datasets covering diverse molecular structures and properties, endowing the model with enhanced generalization capabilities. BERT adapts to various compound types—including organic compounds, inorganic compounds, and mixtures—without requiring separate model designs for each category. BERT’s primary model architecture comprises an input representation layer, a multi-layer Transformer Encoder stack, and an output layer. Figure 3 illustrates the core structure of the BERT model.

**Figure 4:**
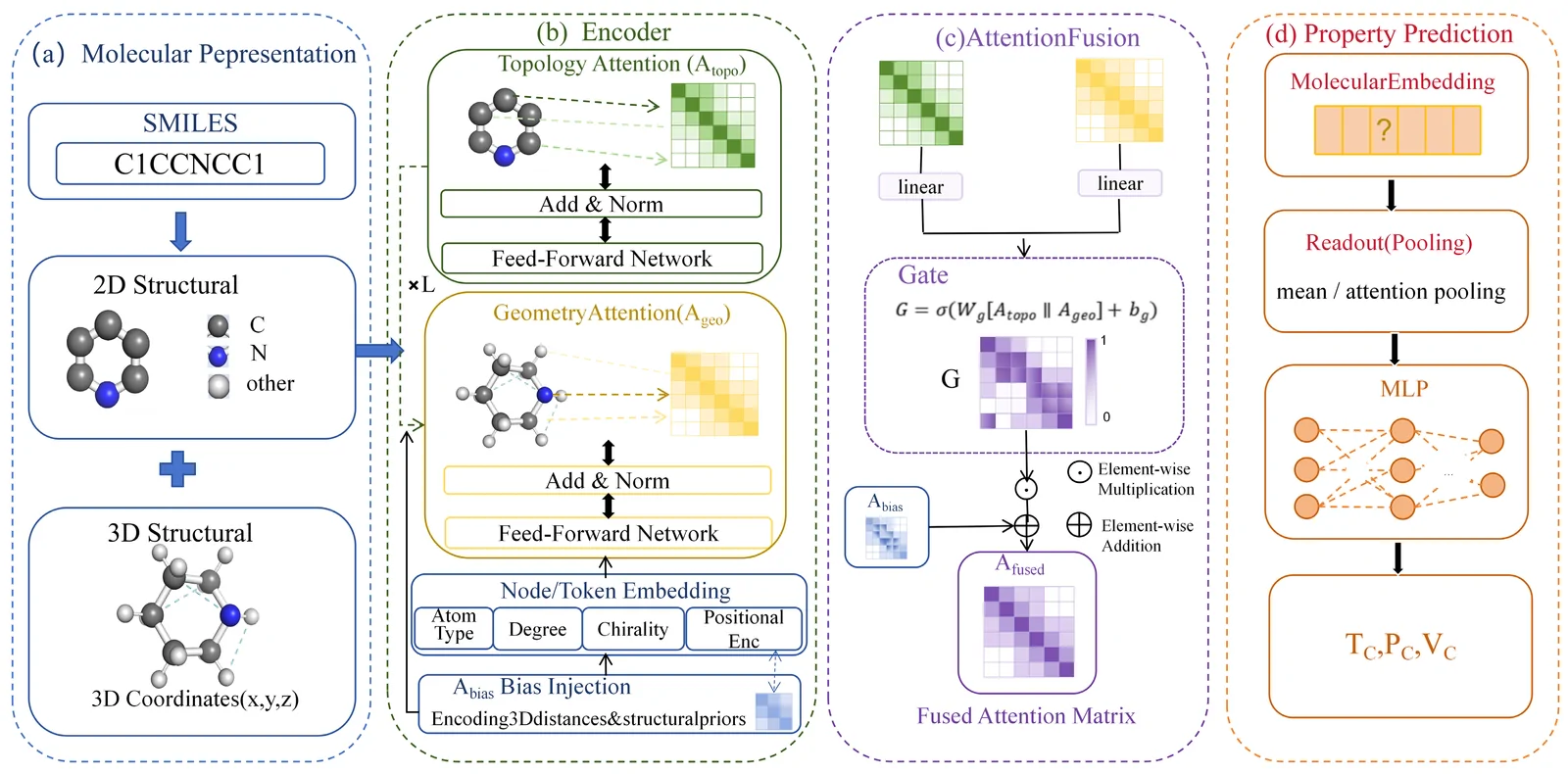
The model comprises four core components.

This study proposes 3DGNN-BERT, a multimodal deep learning framework that collaboratively integrates the semantic information of one-dimensional SMILES sequences, the topological structure of two-dimensional molecular graphs, and three-dimensional spatial geometric information to achieve accurate prediction of molecular critical properties. As shown in Figure 4, the model comprises four core components: a sequence encoder, a geometric encoder, an adaptive fusion module, and a property prediction head.

Given an input triple (x, A, C): $x\in Z^{L}$ is a sequence of atomic-type indices (containing the <global> marker; L is the length of the sequence), $A\in \{0,1{\}}^{L\times L}$ is an adjacency matrix (encoding chemical bond connections, non-bonded positions are masked as −109), $C\in R^{L\times 3}$ is the atomic 3D coordinates.

#### 2.3.1. Sequence–Topology Joint Encoder

The sequence–topology encoder is based on a Transformer architecture with (N = 6) encoder layers, model dimension $d_{model}$ = 256, and h = 8 attention heads. Tokenized SMILES representations are embedded into $d_{model}$-dimensional vectors and combined with the corresponding sequence-level token features.

Standard self-attention does not explicitly encode covalent connectivity. Therefore, a SMILES-derived molecular adjacency matrix is incorporated as an additive structural bias in the attention logits:

(1) $$\begin{array}{c} \mathrm{Attention}(Q,K,V):=\mathrm{softmax}(\frac{QK^{⊤}}{\sqrt{d_{k}}}+A_{\mathrm{bias}})V \end{array}$$

where $A_{bias,ij}=0$ for bonded atom pairs (and self-pairs) and $A_{bias,ij}=-10$ for non-bonded pairs. Thus, non-bonded atom pairs are strongly down-weighted during attention calculation while information exchange through multiple Transformer layers remains possible.

For each attention head,

(2) $$\begin{array}{c} Q=XW^{Q},K=XW^{K},V=XW^{V} \end{array}$$

and the outputs of all heads are concatenated and linearly projected. Each encoder layer further contains a two-layer feed-forward network with GELU activation:

(3) $$\begin{array}{c} FFN(H)=GELU(HW_{1}^{\left(l\right)}+b_{1}^{\left(l\right)})W_{2}^{\left(l\right)}+b_{2}^{\left(l\right)} \end{array}$$

Residual connections and layer normalization are applied after the multi-head attention and feed-forward sublayers:

(4) $$\begin{array}{c} Z^{\left(l\right)}=LayerNorm(H^{\left(l\right)}+MHA(H^{\left(l\right)})) \end{array}$$

(5) $$\begin{array}{c} H^{\left(l+1\right)}=LayerNorm(Z^{\left(l\right)}+FFN(Z^{\left(l\right)})) \end{array}$$

#### 2.3.2. Geometric Encoder

The geometric encoder uses atomic coordinates only through rotation- and translation-invariant interatomic distances. For a molecule containing L atoms with coordinate matrix $C\in R^{L\times 3}$, the pairwise Euclidean distance matrix is calculated as

(6) $$\begin{array}{c} D_{ij}=\sqrt{\sum_{k=1}^{3}{\left(C_{ik}-C_{jk}\right)}^{2}+\varepsilon }\; \end{array}$$

where $\varepsilon =10^{-8}$ is included for numerical stability.

Rather than directly projecting Cartesian coordinates, an invariant four-dimensional feature vector is computed for each atom:

(7) $$\begin{array}{c} f_{i}=[\mu_{i},\sigma_{i},d_{i}^{min},d_{i}^{max}] \end{array}$$

where $\mu_{i}$, $\sigma_{i},\;\;d_{i}^{min}$ and $d_{i}^{max}$ denote the mean, standard deviation, minimum, and maximum distances from atom i to all valid non-self atoms, respectively. These features are projected into a $d_{geo}=128$-dimensional node representation.

The geometric branch consists of two GeometricGNN layers, each containing a distance-aware attention operation and a distance-aware message-passing operation, followed by residual connections and layer normalization. The resulting geometric representation is projected to $d_{model}$ = 256 dimensions and aligned with the atom-level representation of the sequence–topology branch.

In the present implementation, pairwise Euclidean distances are used as geometric inputs. No explicit bond-angle, dihedral-angle, van der Waals-energy, radial-basis-expansion, or cutoff-based neighbor feature is included.

#### 2.3.3. Feature Fusion and Property Prediction

The sequence–topology branch produces atom-level representations $H_{\mathrm{top}}\in R^{L\times d_{\mathrm{model}}}$, whereas the geometric branch produces $H_{\mathrm{geo}}\in R^{L\times d_{\mathrm{geo}}}$. To align the two modalities, the geometric representation is first projected to the sequence-model dimension:

(8) H^geo=HgeoWalign+balign

where $W_{\mathrm{align}}\in R^{d_{\mathrm{geo}}\times d_{\mathrm{model}}}$. The topology and geometry representations are aligned at the atom level. A feature-wise gate is then computed from their concatenation:

(9) Z=[Htop‖H^geo]∈RL×2dmodel,G=σ(ZWg+bg)∈RL×dmodel

where $W_{g}\in R^{2d_{\mathrm{model}}\times d_{\mathrm{model}}},b_{g}\in R^{d_{\mathrm{model}}}$, and σ(·) is the sigmoid activation function. The gate provides an atom- and feature-dependent weighting between topological and geometric representations.

(10) Hfuse=[G⊙Htop+(1−G)⊙H^geo]Wf+bf

where $W_{f}\in R^{d_{\mathrm{model}}\times d_{\mathrm{model}}},b_{f}\in R^{d_{\mathrm{model}}}$, and ⊙ denotes element-wise multiplication. The gate is used as a learnable feature-wise fusion operation and is not presented as a standalone novel attention mechanism.

A molecular representation is obtained by masked mean pooling over valid atom tokens:

(11) $$\begin{array}{c} h_{mol}=\frac{\sum_{i=1}^{L}m_{i}h_{fuse,i}}{max(\sum_{i=1}^{L}m_{i},1)} \end{array}$$

where $m_{i}=1(validatom)$ for a valid atom token and $m_{i}=0(padding)$ for padding tokens. The pooled representation is passed to a two-layer multilayer perceptron for property prediction:

(12) y^=W2Dropout(LeakyReLU(W1hmol+b1))+b2

#### 2.3.4. Conformer Source and Rotational-Invariance Assessment

The three-dimensional coordinates used in this study were obtained from the SDF records downloaded from the PubChem database for the corresponding compounds. For each molecule, one deposited PubChem conformer was used as the input geometry. No conformers were generated using ETKDG, and no additional MMFF94 or UFF geometry optimization, conformer ensemble generation, energy ranking, or representative-conformer selection was performed in the present work. Therefore, the reported 3D results should be interpreted as predictions based on the available deposited PubChem conformations rather than on conformationally averaged molecular properties.

To examine the rotational behavior of the geometric representation, a random-rotation inference experiment was conducted using the held-out test set for critical-temperature prediction (seed 27, fold 4). Each molecular coordinate set was transformed by 20 independently sampled three-dimensional rotation matrices, while atom types, molecular connectivity, model weights, and all other inputs were kept unchanged. The predictions from the rotated structures were compared with those obtained from the original coordinates. The mean absolute difference between rotated and original predictions was 3.56 × 10^−4^ K, and the maximum absolute difference was 0.0253 K. These deviations are negligible relative to the model test MAE (52.71 K), indicating that the implemented distance-based geometric representation is numerically invariant to global rigid rotations

This assessment concerns rotational invariance only. The effect of conformer choice remains a limitation of the present study because a single deposited conformer was used for each compound. Future work should evaluate conformer ensembles, conformational averaging, and standardized geometry-generation and optimization protocols, particularly for flexible molecules and critical-volume prediction.

### 2.4. Training Strategies

During the pretraining phase, the model uses masked atom prediction as a self-supervised task to learn a general-purpose chemical representation of molecules. Specifically, for each input molecule, a masked subset M is randomly sampled from the atom set V with probability *p* = 0.15; for each masked atom i ∈ M, its atomic type embedding is replaced with a learnable <mask> token, while retaining its 3D coordinates xi and topological connectivity with other atoms to ensure the integrity of geometric and structural context. The model’s objective is to predict the true element type ci of the masked atom based on the topological neighborhood and spatial configuration of the surrounding unmasked atoms. This task is equivalent to a multi-class classification problem in the element category space, with the optimization objective of minimizing the cross-entropy loss:

(13) $$\begin{array}{c} L_{\mathrm{pre}}=-\frac{1}{\left|M\right|}\underset{i\in M}{\sum }\log P\left(x_{i}\right) \end{array}$$

After pretraining is completed, when applying the model to specific tasks, fine-tuning can be used to further optimize model parameters, enabling better adaptation to task characteristics and improving prediction accuracy. The core of this research lies in effectively transferring the general molecular representations learned during the pretraining phase to the specific regression task of predicting critical properties of compounds through fine-tuning techniques. The fine-tuning process does not simply employ the pre-trained model as a fixed feature extractor. Instead, it involves further supervised training on a small-scale, high-quality labeled dataset for the target task, precisely optimizing the model’s representational capabilities for critical property prediction. The specific fine-tuned pre-training parameter settings are shown in Table 1. The 28-word vocabulary list is shown in Table 2.

### 2.5. Model Evaluation and Validation

For the regression task, this study selected Mean Squared Error (MSE), Mean Absolute Error (MAE), and Coefficient of Determination. Mean Squared Error (MSE):

(14) $$\begin{array}{c} \mathrm{MSE}=\frac{1}{n}\sum_{i=1}^{n}{\left(y_{i}-{\hat{y}}_{i}\right)}^{2} \end{array}$$

Among these, yi represents the actual value, ${\hat{y}}_{I}$ denotes the predicted value, and n indicates the number of samples. MSE measures the average squared difference between predicted and actual values, with smaller values indicating better model performance.

Mean Absolute Error (MAE):

(15) $$\begin{array}{c} MAE=\frac{1}{n}\sum_{i=1}^{n}\left|y_{i}-{\hat{y}}_{i}\right| \end{array}$$

MAE measures the average absolute difference between predicted and actual values and is less sensitive to outliers.

Coefficient of Determination (R^2^):

(16) $$\begin{array}{c} R^{2}=1-\frac{\sum_{i=1}^{n}{\left(y_{i}-{\hat{y}}_{i}\right)}^{2}}{\sum_{i=1}^{n}{\left(y_{i}-\overset{‾}{y}\right)}^{2}} \end{array}$$

R^2^ indicates the extent to which a model explains the variation in data, with values ranging from 0 to 1. The closer the value is to 1, the better the model’s performance.

Given that the dataset for the compound’s critical properties contains only a few hundred entries, this study selected K-fold cross-validation as more suitable for small datasets. The original dataset was randomly shuffled to ensure randomness in data distribution. The shuffled dataset was then divided into K mutually exclusive subsets (referred to as “folds”). Typically, K is set to 5 or 10. In this study, K was chosen as 5. The size of each subset is approximately 1/K of the total sample.

To verify whether the trained atomic embeddings capture chemical semantic information, we performed t-SNE dimensionality reduction visualization on the atomic representations of the untrained and trained models.

**Figure 5:**
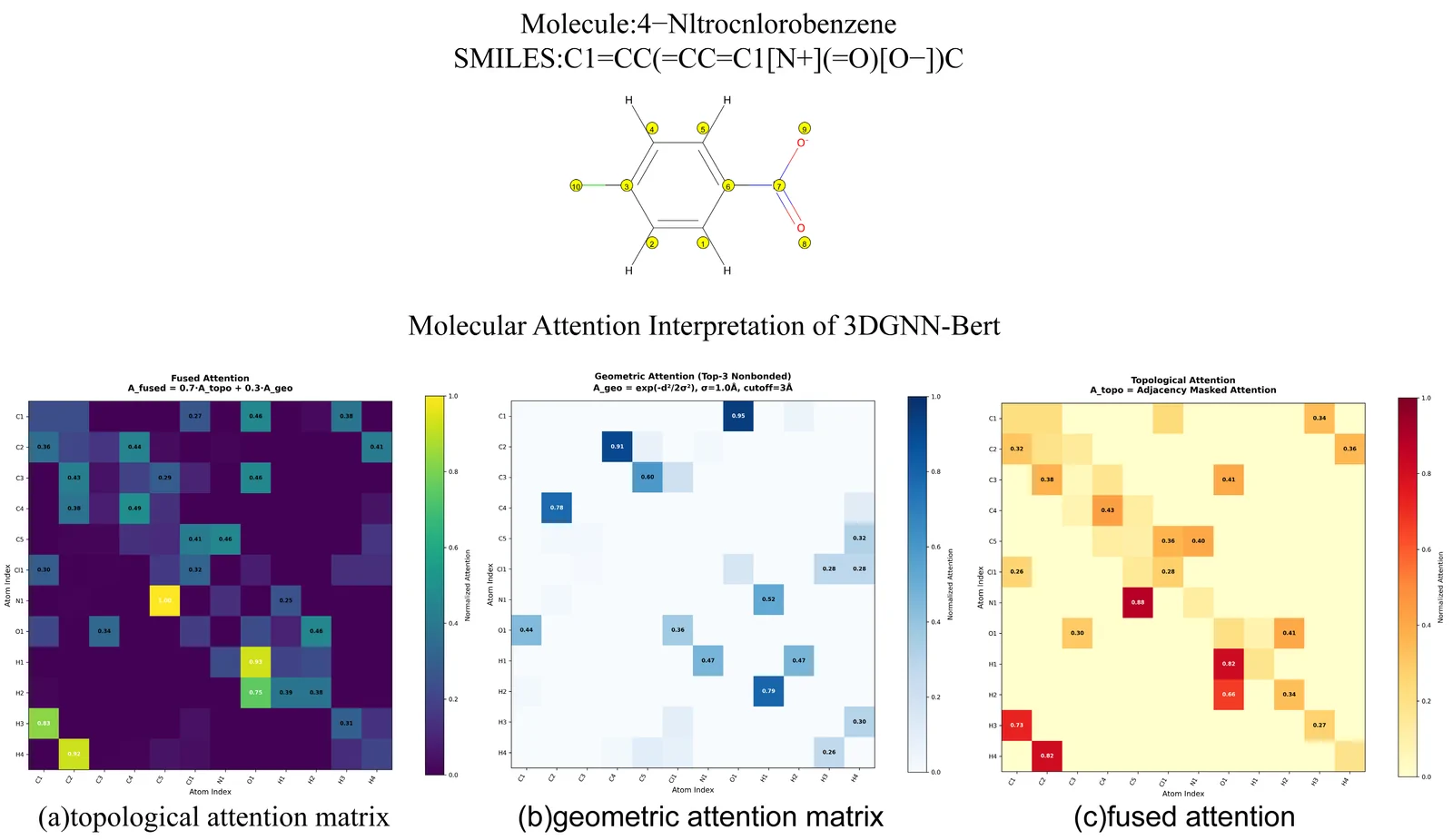
4-nitrochlorobenzene molecule in the 3DGNN-BERT model.

Figure 5 illustrates the attention interpretability analysis of the 4-nitrochlorobenzene molecule in the 3DGNN-BERT model. Figure 5a shows the topological attention matrix, where high weights are concentrated on atom pairs connected by chemical bonds (e.g., C1-C2, C3-Cl1), confirming that the graph-biased attention mechanism effectively encodes the molecular skeleton; Figure 5b shows the geometric attention matrix, where high weights reflect non-bonded interactions between spatially adjacent atoms (e.g., O1-H2, Cl1-H3), indicating that GeometricGNN successfully captures 3D steric effects; Figure 5c shows the fused attention, which integrates bond connectivity and spatial proximity; for example, cross-functional group attention between nitro oxygen atoms (O1, O2) and benzene ring hydrogen atoms (H1–H4) is significantly activated. These results demonstrate that the model can distinguish and fuse two types of heterogeneous information—topological connectivity and geometric proximity—providing an interpretable attention mechanism for predicting critical properties.

## 3. Results and Discussion

During training, accuracy and loss rates of all three models exhibited distinct patterns across stages, which were systematically validated in this study. The core differences between the Small, Medium, and Large Transformer configurations lie in the trade-offs between computational efficiency, expressive power, and resource requirements, each with clear suitability for specific scenarios. The advantage of small models lies in their extreme lightness and efficiency. With minimal parameters, they train and infer extremely fast, making them suitable for small datasets with low computational costs and minimal time requirements. Medium models excel in cost-effectiveness and versatility. They strike the optimal balance between performance and resource consumption, achieving enhanced training accuracy. Large models excel in top-tier expressive power and predictive accuracy. They learn rapidly but demand high memory resources and require extremely long training times. They are unsuitable for small datasets and prone to overfitting. Since the data in this study peaks at 911 instances, it is better suited for training in small and medium modes. Therefore, more extensive training adjustments were conducted in these two modes, while fewer and smaller-scale training sessions were performed in the large mode.

### 3.1. Pre-Training Results

Pre-training with a threshold temperature yielded the best results in the 3D-GNN model, which takes SMILES and three-dimensional molecular geometry as input. In the Medium mode, 1000 cycles of pre-training resulted in an accuracy of 0.9877 and a loss of 0.0023. Regarding the critical pressure task, the 3D-GNN model that uses SMILES and three-dimensional molecular geometry diagrams as inputs achieved the best pre-training results. In the Medium mode, its accuracy reached 0.9931. In the critical volume task, the 3D-GNN model, which also takes SMILES and three-dimensional molecular geometry graphs as inputs, achieved the optimal pre-training performance—with an accuracy of 0.9931 and a loss of 0.006 in the 1200-epoch medium model. The accuracy and loss rates for critical properties across the three models’ different cycles are shown in Table 3 and Table 4. These metrics were monitored on the pre-training validation set to determine the optimal checkpoint for subsequent fine-tuning on the downstream critical property prediction tasks.

Overall, when predicting critical properties, the accuracy of the pre-trained models improved significantly as the number of parameters increased. The Medium model achieved a significantly higher accuracy than the small model under optimal conditions, indicating that more complex model architectures possess stronger representational learning capabilities and can more accurately capture the complex relationships between structure and critical properties. Among these, the 3D-GNN model performed the best.

2D structural diagrams rely solely on topological connectivity and involve calculations in “distance space”; they are invariant under translation and rotation but cannot distinguish between enantiomers, and their information is limited to interatomic bonding paths and chemical bond types. In contrast, three-dimensional molecular geometry diagrams are based on real-space coordinates and include precise quantifications of physically observable properties such as molecular shape, volume, surface area, and charge distribution [43]; they can distinguish between molecules that have the same topology but different spatial configurations. For the prediction of critical properties, three-dimensional molecular geometry diagrams have a significantly greater impact than 2D structural diagrams. This is because critical properties are bulk thermodynamic properties that directly depend on the packing efficiency of molecules in three-dimensional space, surface interactions, and the spatial distribution of intermolecular forces—all of which require precise geometric information [44].

For the critical temperature and critical pressure tasks, the Medium-sized model reached its peak performance after approximately 1000 training epochs, while for the critical volume task, it reached its peak after 1200 epochs; its overall performance was superior to that of the small and large models. Although the Large model converges faster, it is not suitable for small datasets and is prone to overfitting; the small model, on the other hand, lacks sufficient representational capacity. Therefore, the 3DGNN-BERT model at the medium scale achieved the best accuracy and the lowest loss rate during pre-training.

### 3.2. Evaluation of Downstream Tasks for Models

Across all model scales, the 3DGNN-BERT model demonstrates the strongest or near-strongest overall prediction performance. Particularly at the most representative medium scale, 3DGNN-BERT achieved the best performance among all models. This result fully demonstrates that incorporating the three-dimensional spatial geometry of molecules into a multimodal learning framework plays a decisive role in enhancing the accuracy of critical temperature prediction. The specific values of the evaluation metrics for critical properties, including R^2^, MAE, RMSE and MSE, are presented in Table 5.

To assess the utility of augmented structural descriptors for conventional machine-learning baselines, random forest (RF) and support vector regression (SVR) models were evaluated using two input schemes: Morgan fingerprints alone and Morgan fingerprints supplemented with eight structure-derived descriptors. The baseline representation consisted of a 2048-bit Morgan fingerprint generated from the SMILES string. The augmented representation additionally included molecular weight, MolLogP, topological polar surface area, radius of gyration, asphericity, eccentricity, plane-of-best-fit (PBF), and inertial shape factor. Among these descriptors, radius of gyration, asphericity, eccentricity, PBF, and inertial shape factor characterize molecular size, three-dimensional shape, and conformational geometry.

The augmented descriptor representation improved the performance of the SVR model for critical temperature and critical pressure, whereas critical volume exhibited a decline. For SVR, the R^2^ values for temperature, pressure, and volume changed from 0.5244, 0.3823, and 0.8856 to 0.5870, 0.5457, and 0.7338, respectively. The corresponding MAE values changed from 0.0593, 0.0779, and 0.0426 to 0.0442, 0.0694, and 0.0793, while the MSE values changed from 0.0067, 0.0412, and 0.0050 to 0.0058, 0.0303, and 0.0117, respectively.

RF showed substantial improvements for critical temperature and critical volume after descriptor augmentation, while critical pressure remained essentially unchanged. The R^2^ values for temperature, pressure, and volume changed from 0.6333, 0.3186, and 0.4817 to 0.8381, 0.3160, and 0.8745, respectively. The corresponding MAE values changed from 0.0473, 0.0756, and 0.1093 to 0.0209, 0.0759, and 0.0407, and the MSE values changed from 0.0051, 0.0455, and 0.0228 to 0.0023, 0.0456, and 0.0028, respectively.

These results indicate that the augmented structural descriptor set provides complementary information beyond Morgan fingerprints alone. In particular, the improved performance of RF+3D and SVR+3D supports the practical value of incorporating structural and conformational information into critical-property prediction. However, because the augmented representation contains both conventional physicochemical descriptors and 3D shape descriptors, this comparison should be interpreted as evidence for the utility of the combined descriptor set rather than as an isolated quantification of the contribution of 3D geometry alone.

As shown in Table 5, 3DGNN-BERT consistently outperforms SVR and RF across most metrics, confirming that expressive architectures are required to fully exploit rich 3D graph representations. All R^2^, MAE, and MSE values in this comparison were calculated in the log10-transformed target space. These controlled comparisons demonstrate that identical 3D graph embeddings yield markedly different outcomes depending on the readout architecture. The gains of 3DGNN-BERT thus stem from its capacity to jointly learn geometric, topological, and semantic representations through differentiable message passing and chemical language pretraining, rather than from input features alone.

Critical pressure exhibited a consistently lower coefficient of determination than critical temperature and critical volume across all the evaluated models. Moreover, incorporating the 3D structural representation did not substantially remove this difference, indicating that it cannot be attributed solely to the molecular-input dimensionality. Critical pressure is thermodynamically related to critical temperature, critical volume, and the critical compressibility factor through $P_{c}=Z_{c}RT_{c}/V_{c}$. It therefore reflects the combined influence of molecular size, cohesive interactions, packing, and non-ideal fluid behavior. Factors such as polarity, hydrogen-bond association, polarizability, molecular flexibility, and the acentric factor may not be completely encoded by a 2D molecular image or a single static 3D conformer. In addition, the critical-pressure data were obtained from heterogeneous sources, and the data-quality assessment identified several records with potentially inconsistent units or decimal placement. Such label uncertainty affects all models independently of their input representation. These factors collectively provide a plausible explanation for the lower critical-pressure prediction accuracy.

**Figure 6:**
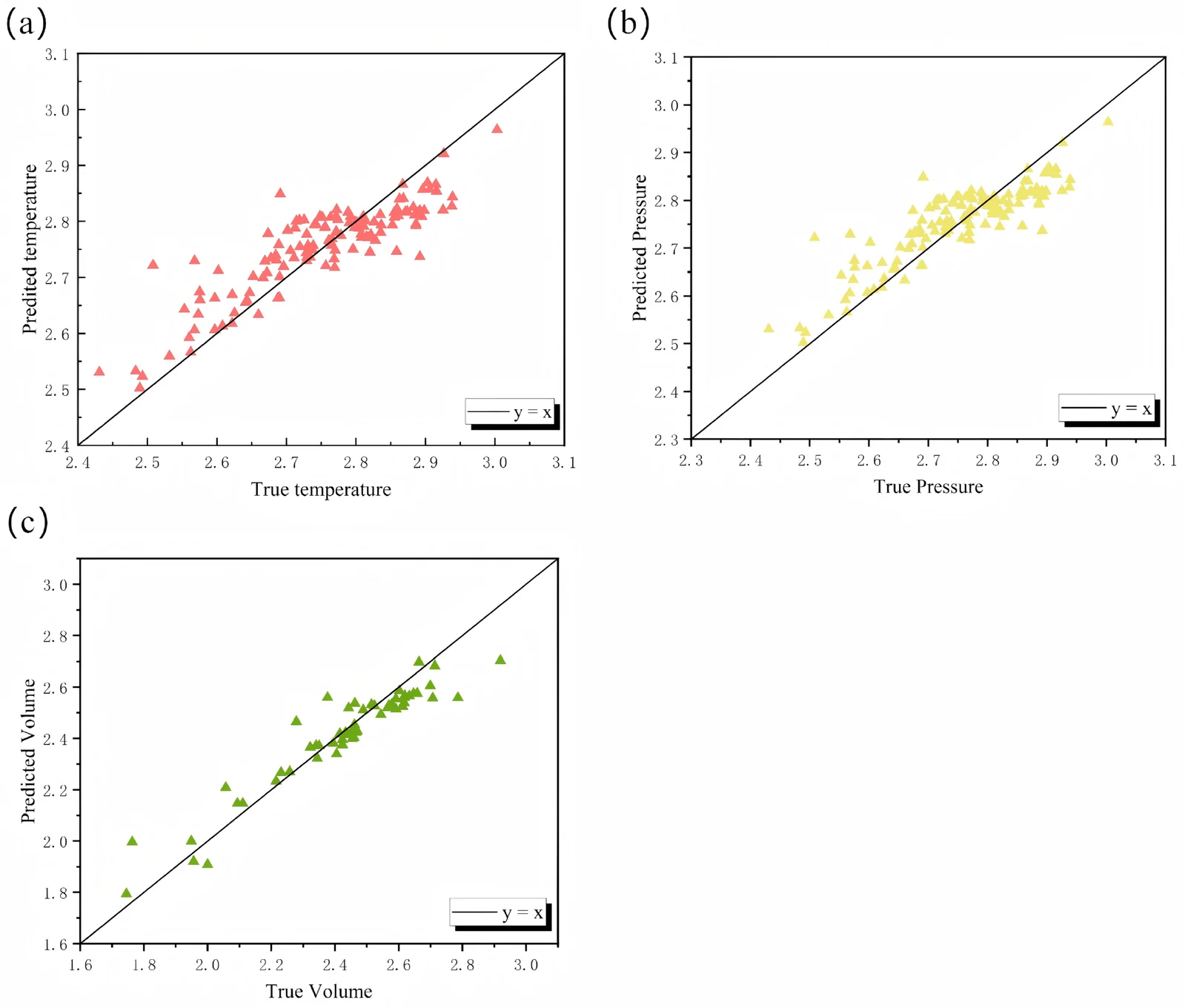
Scatter plots: (**a**) observed and predicted critical temperatures; (**b**) observed and predicted critical pressures; (**c**) observed and predicted critical volumes.

Figure 6 shows the scatter plots of the predicted critical temperatures by the optimal models for critical temperature, critical pressure, and critical volume against the actual experimental values on the test set. The dotted diagonal line represents the ideal prediction line (y = x). As shown in the figure, the predicted data are tightly clustered on both sides of the diagonal, demonstrating a strong linear correlation trend within the evaluation range without any significant systematic bias. This visualization intuitively corroborates the high R^2^ values and low MAE and MSE values reported in Table 5, providing both quantitative and qualitative confirmation of the accuracy and reliability of this model’s predictions.

To better understand the limitations of the coupled Bert–GNN model, we examined the compounds exhibiting the largest absolute deviations in Figure 6. For critical temperature (Tc), the most significant outliers are ethylene carbonate, 2-ethyl-1-butanol, and isophthalic acid. All three are strong hydrogen-bond donors or acceptors, yet the current graph construction only encodes covalent bonds; hydrogen-bonding interactions are entirely absent from both the 2D adjacency matrix and the 3D distance-based edge features. Consequently, the GNN cannot learn the extra cohesive energy arising from intermolecular hydrogen-bond networks, leading to systematic underprediction of Tc for this chemical class. In the case of isophthalic acid, the issue is compounded by the scarcity of high-quality experimental data: its melting point (347 °C) exceeds the accessible boiling range, and the reported critical temperature is derived from high-temperature sealed-tube extrapolations with large inherent uncertainty.

For critical pressure (Pc), the largest deviations are observed for cyanuric chloride, p-xylene, and 1,2,3,4-tetrachlorobenzene. A common feature of these compounds is high geometric symmetry (D_3_h for cyanuric chloride, D_2_h for p-xylene), which is not preserved by canonical SMILES tokenization. The Bert encoder processes p-xylene as an asymmetric branched string (CC1=CC=C(C=C1)C), obscuring the underlying symmetry and causing the transformer to overestimate structural complexity. Meanwhile, the 3D GNN branch provides limited assistance because these essentially planar molecules collapse into near-degenerate 2D graphs, reducing the discriminative power of geometric edge features. The model therefore falls back to SMILES-driven predictions, losing the geometric information necessary to capture the reduced vaporization entropy associated with high symmetry.

Critical volume (Vc) shows the most extreme outliers: nonylcyclopentane, nitric oxide, and 3,5-dimethylphenol. Nonylcyclopentane is a highly flexible molecule with a long alkyl tail attached to a cyclopentane ring. Our pipeline generates a single low-energy conformer, but at temperatures approaching the critical point (~717 K), the actual molecular ensemble samples a broad distribution of conformations; a frozen 3D graph therefore misrepresents the effective molecular volume. Nitric oxide presents the opposite problem: as a diatomic inorganic radical with only two meaningful SMILES tokens, the Bert encoder produces degenerate embeddings, while its partial dimerization (2NO ⇌ N2O2) near the critical point introduces behavior that lies far outside the training distribution. 3,5-Dimethylphenol, like the Tc outliers, suffers from missing hydrogen-bond edges in the graph representation, which alters the predicted liquid-phase packing density.

These patterns suggest that the largest deviations stem from two sources: (i) representation failures in the current Bert+GNN pipeline, including symmetry-agnostic SMILES tokenization, the absence of non-covalent interaction edges, and reliance on single-conformer 3D graphs; and (ii) experimental uncertainty for compounds that are either high-melting solids, highly reactive, or poorly characterized in the literature. Addressing the former will require symmetry-aware SMILES augmentation, dynamic hydrogen-bond edge insertion based on 3D donor–acceptor geometry, and ensemble averaging over multiple conformers for flexible molecules

#### Stratified Independent Test-Set Validation

To rigorously assess the generalization capability of the proposed model, a stratified independent test-set validation was conducted under a data-partitioning scheme designed to eliminate data leakage. The full dataset was first sorted by target-property values and divided into contiguous intervals for critical temperature, pressure, and volume, respectively. Within each interval, compounds were randomly assigned to training, validation, and test sets at a ratio of 70:15:15. This stratified strategy ensures that the model is evaluated across the complete dynamic range of each property, preventing performance inflation from an uneven distribution of easy-to-predict compounds in any single split.

For the Bert+3D-GNN model, the training set (70%) was used for both the self-supervised pre-training of the Bert encoder and the supervised fine-tuning of the full coupled architecture. The validation set (15%) was employed exclusively for hyperparameter tuning, early-stopping monitoring, and selection of the optimal model weights; no test-set information was accessed during this phase. The test set (15%) was held out entirely until the final evaluation, where the optimized model was applied exactly once to generate the reported metrics. This strict three-way separation guarantees an unbiased estimate of out-of-sample performance and confirms that no data leakage occurred. Table 6 shows the results of the validation experiments using a stratified independent test set.

On the stratified test set for critical temperature, the model achieved strong predictive performance with a coefficient of determination comparable to the internal random-split results, exhibiting only a modest decay in explanatory power. The mean absolute error remained small in absolute terms, while the root-mean-square error increased relative to the internal validation, reflecting the forced inclusion of extreme-interval compounds—particularly those with very high or very low Tc values that are underrepresented in random splits. This pattern indicates that the model generalizes robustly across the full dynamic range of critical temperature. The disproportionate increase in RMSE relative to MAE further suggests that a small subset of chemically challenging compounds drives the residual error.

### 3.3. Baseline Methods for Comparison

To contextualize the predictive performance of the proposed Bert–GNN coupled architecture, three representative baseline methods spanning different methodological paradigms were selected for comparison: a classical group-contribution method (Joback–Reid), a 3D geometry-based deep-learning model (SchNet), and a 2D graph-based message-passing neural network (Chemprop). This tiered selection allows us to isolate the added value of our coupled SMILES–graph representation against (i) traditional additive estimation, (ii) pure 3D geometric learning, and (iii) state-of-the-art 2D topological learning.

Joback–Reid method. As the most widely adopted group-contribution approach for critical-property estimation, Joback–Reid decomposes each molecule into a predefined set of first-order functional groups and linearly sums their contributions. All three critical properties (Tc, Pc, Vc) were predicted using the standard parameter table given by Joback and Reid (1987) [20]. No second-order corrections were applied, ensuring a fair comparison against the first-order capabilities of our model. In this study, the Joback group contribution method was used to predict the critical properties of pure substances, and a systematic evaluation was conducted based on 813 experimental data points for critical temperature, 522 for critical pressure, and 335 for critical volume.

Chemprop employs a directed message-passing neural network in which hidden states are propagated over directed chemical bonds. Its molecular graph is constructed from SMILES, and the input contains atom and bond attributes but no SDF-derived coordinates. SchNet receives atomic numbers and Cartesian coordinates extracted from the PubChem SDF files. Pairwise distances are expanded using radial basis functions and processed through continuous-filter interaction layers. SchNet does not receive SMILES embeddings, Morgan fingerprints, manually calculated molecular descriptors, or the representations generated by the proposed BERT encoder.

The two baselines therefore provide complementary controls: Chemprop evaluates the predictive capability of explicit two-dimensional bond topology, whereas SchNet evaluates the capability of a single-modality geometric representation based on atomic identities and spatial coordinates.

For each target property, all models used the same property-specific training, validation, and test molecules. Target normalization parameters were calculated from the training set only. Predictions were inverse-transformed to physical units before evaluation. Errors for Tc, Pc, and Vc are reported in K, MPa, and cm^3^ mol^−1^, respectively. Each model was trained using three random initialization seeds (42, 123, and 2024).

All baseline models were evaluated using identical metrics (MAE, RMSE, and relative error) and the same train–validation–test data partitions described in Section 3.2. The results of this comparative analysis are presented in Table 7.

For critical temperature (Tc), the proposed model achieves an R^2^ of 0.904, a MAE of 30.17 K, and an RMSE of 41.49 K, substantially outperforming all baselines. Notably, SchNet (R^2^ = 0.484, RMSE = 99.04 K) and Chemprop (R^2^ = 0.560, RMSE = 91.53 K) both struggle with this property, suggesting that neither pure 3D geometry nor 2D topology alone captures the complex intermolecular interactions governing Tc. Surprisingly, the classical Joback method (R^2^ = 0.763, RMSE = 65.49 K) outperforms both deep-learning baselines, underscoring that additive group contributions retain predictive power for temperature-related properties, though they still fall significantly short of the coupled model.

For critical pressure (Pc_)_, the ranking shifts. Joback delivers the lowest MAE (0.412 MPa), likely because Pc correlates strongly with molecular size and atom counts, which group-contribution methods estimate robustly. However, Joback’s R^2^ (0.632) remains lower than that of our model (0.757), indicating that the coupled architecture better explains the variance in the data despite a slightly higher absolute error. SchNet again performs the poorest (R^2^ = 0.628, RMSE = 1.167 MPa), reinforcing the observation that static 3D conformers provide limited information for pressure-related properties.

For critical volume (Vc), our model achieves an R^2^ of 0.891 and an RMSE of 47.01 cm^3^·mol^−1^, closely followed by Chemprop (R^2^ = 0.867). Joback exhibits the smallest MAE (16.27 cm^3^·mol^−1^) and a competitive R^2^ (0.883), confirming that molecular volume is inherently well-described by additive atomic contributions. Nevertheless, the coupled model’s superior R^2^ indicates that it generalizes better across structurally diverse compounds, particularly those with non-additive steric or hydrogen-bonding effects.

While Joback remains surprisingly competitive for Pc and Vc, it fails to match the coupled model’s accuracy for Tc and its overall explanatory power (R^2^). More importantly, the poor performance of SchNet highlights a key limitation of pure 3D approaches for critical-property prediction: critical properties reflect ensemble-averaged intermolecular behavior rather than single-molecule quantum states, making 3D coordinate-based representations suboptimal without complementary topological or sequential information. The consistent superiority of the Bert–GNN coupling across all metrics demonstrates that integrating SMILES-based sequential semantics with 2D/3D graph representations is essential for advancing beyond both classical additive methods and unimodal deep-learning alternatives.

### 3.4. Atom Embedding Visualization

**Figure 7:**
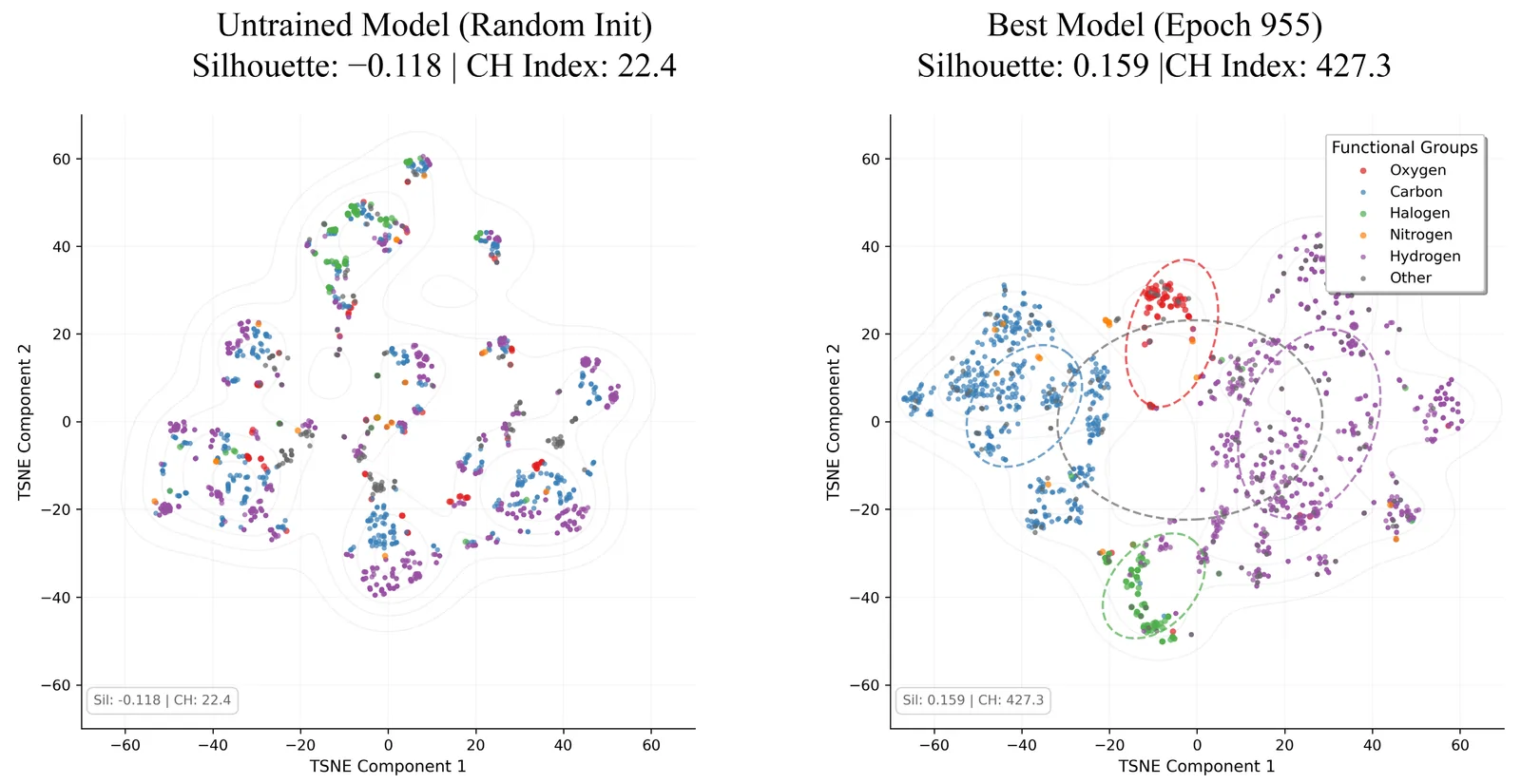
t-SNE visualization of atom embeddings obtained from the untrained model (**left**) and the 3DGNN-BERT model trained on the critical property regression task (**right**).

Figure 7 presents t-SNE visualizations of atom-level embeddings extracted from the untrained model (left) and the trained 3DGNN-BERT model (right), with points colored by functional group categories. The untrained model exhibits an approximately uniform distribution with highly overlapping functional groups (Silhouette = −0.118, CH Index = 22.4), indicating that random initialization has not yet encoded chemically meaningful structural distinctions. No discernible clustering pattern emerges; atoms of different chemical identities are intermingled throughout the embedding space.

In contrast, the trained model shows distinctly structured clustering (Silhouette = 0.159, CH Index = 427.3). Atoms bearing similar functional groups aggregate into compact local clusters—Carbon (blue, upper left), Hydrogen (purple, right), Halogen (green, lower)—while distinct groups occupy well-separated regions. Notably, the Nitrogen and Oxygen substituents form tight subclusters within broader groupings, reflecting the model’s sensitivity to heteroatom electronic effects. This emergent organization demonstrates that the model successfully learns chemically interpretable representations under regression supervision, encoding functional group identity without explicit architectural bias toward such categorization.

The comparison confirms that training transforms an unstructured embedding space into one characterized by functional group organization. This chemically meaningful representation foundation directly supports accurate critical property prediction, as structurally similar molecules naturally cluster in the learned embedding space.

**Figure 8:**
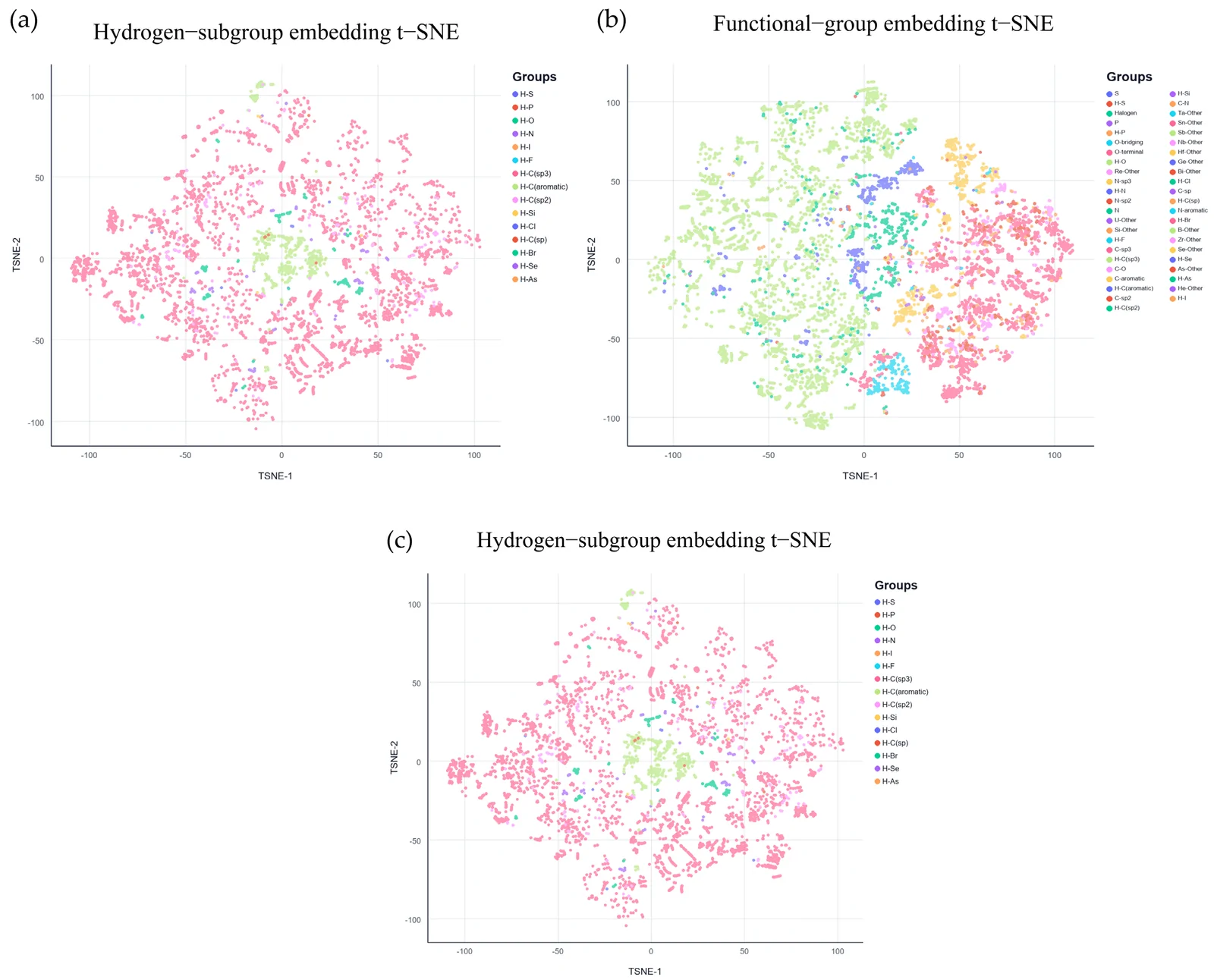
t-SNE visualization of the learned atomic representations according to (**a**) atom type, (**b**) local functional-group environment, and (**c**) hydrogen-containing subgroups.

To further examine whether the learned representation distinguishes different local chemical environments, the hydrogen atoms were disaggregated according to the atom to which they are bonded and its bonding state. The resulting subgroups include H–C(sp^3^), H–C(sp^2^), H–C(aromatic), H–C(sp), H–O, H–N, H–S, and several less frequent hydrogen environments. As shown in Figure 8, hydrogen atoms do not form a single homogeneous cluster in the learned embedding space. H–C(sp^3^), which is the most abundant subgroup in the dataset, occupies a relatively broad region, consistent with the structural diversity of aliphatic carbon environments. H–C(aromatic) and H–C(sp^2^) exhibit more localized distributions, while hydrogen atoms bonded to heteroatoms, particularly H–O and H–N, tend to occupy regions distinguishable from those of carbon-bound hydrogen. These results suggest that the model does not represent hydrogen atoms solely according to their elemental identity but also encodes information related to hybridization, neighboring atoms, and the local bonding environment.

Some overlap between the hydrogen subgroups remains. This is expected because atoms assigned to the same functional-group category may still occur in substantially different molecular environments, while chemically related categories can share similar local structural features. In addition, the dataset is strongly imbalanced, with H–C(sp^3^) being much more abundant than heteroatom-bound and other uncommon hydrogen subgroups. Consequently, the locations and apparent separation of sparsely represented groups should be interpreted cautiously. Furthermore, t-SNE is a nonlinear dimensionality-reduction method that emphasizes local neighborhood relationships; therefore, the apparent distances between clusters do not directly represent quantitative chemical similarity or predictive accuracy. The visualization should thus be regarded as qualitative evidence that the learned features capture chemically meaningful structural differences.

## 4. Conclusions

This study systematically demonstrates the complementary and irreplaceable roles of molecular sequences, two-dimensional topology, and three-dimensional geometric information in predicting critical thermodynamic properties. Ablation experiments show that the critical temperature is highly sensitive to both SMILES sequences and three-dimensional geometry, the critical volume depends almost entirely on graph structure information, and the critical pressure requires the synergy of all three to overcome the prediction bottlenecks inherent in single-modal approaches. Comparisons with the classical group contribution method (Joback–Reid) and mainstream deep learning baselines (Chemprop, SchNet) further confirm that the coupled architecture is comprehensively superior in explanatory power and predictive accuracy, with particularly significant advantages when handling non-additive interactions such as hydrogen bonding, symmetry, and polyhalogenation. At the same time, pressure prediction remains a common challenge across all current methods, with errors primarily stemming from intermolecular association, heterogeneity in experimental data, and the inherent limitations of isolated-molecule representations.

We acknowledge that a single PubChem conformer cannot fully represent the conformational ensemble of a flexible molecule in the liquid state. The employed structure should therefore be regarded as a standardized structural proxy rather than the unique thermodynamic conformation at the critical point. This approximation may be particularly important for long-chain hydrocarbons and other flexible species, whose distance matrices can vary considerably between conformations. Consequently, volumetric properties that depend on molecular packing and accessible molecular volume may be affected.

Although the BERT+3D-GNN model produced a marked improvement in critical-temperature prediction, its statistical performance should not be interpreted as demonstrating readiness for direct industrial application. In particular, the relatively low R^2^ and comparatively large error for critical pressure indicate considerable uncertainty for individual compounds. Critical properties are fundamental inputs to equations of state, phase-equilibrium calculations, equipment design, and the determination of supercritical operating conditions. Prediction errors may therefore propagate into calculated densities, fugacities, phase boundaries, and process-design parameters, especially when a process operates close to the critical region.

At its current level of development, the proposed model is more suitable for preliminary screening, comparison of candidate compounds, and estimation of missing values when experimental data are unavailable. Its predictions should subsequently be verified using reliable experimental measurements, established property databases, or validated thermodynamic methods. The model is not intended to replace experimentally validated critical constants in safety-critical calculations, detailed process simulation, equipment sizing, or plant operation.

Consequently, the proposed contribution is not merely the inclusion of three-dimensional coordinates, but the adaptive integration of complementary molecular representations for property-dependent thermodynamic prediction.

## Figures and Tables

**Table 1:** Pre-training Parameter Settings.

Name	Layer	Head	Embedding Size	Learning Rate	Vocabulary Size
Small	3	6	128	1 × 10^−4^	28
Medium	6	8	256		
Large	12	12	576		

**Table 2:** 28-word vocabulary.

No.	Token	No.	Token	No.	Token	No.	Token
1	<pad>	2	H	3	C	4	N
5	O	6	F	7	S	8	Cl
9	P	10	Br	11	B	12	I
13	Si	14	Se	15	<unk>	16	<mask>
17	<global>	18	Te	19	As	20	Al
21	Zn	22	Ca	23	Ag	24	Mg
25	K	26	Na	27	Fe	28	Cu

**Table 3:** Accuracy of critical properties in three models.

Task	Mode	3DGNN-BERT	2DGNN-BERT	BERT
Critical Temperature (1000 epochs)	Small	0.9143	0.9280	0.8014
	Medium	**0.9877**	0.9385	0.9138
Critical Pressure (1000 epochs)	Small	0.9716	0.9332	0.6509
	Medium	**0.9931**	0.9225	0.8461
Critical Volume (1200 epochs)	Small	0.9870	0.9248	0.7768
	Medium	**0.9890**	0.9157	0.8561

Numbers in bold indicate the best results among the three models.

**Table 4:** Loss rate at critical properties in three models.

Task	Mode	3DGNN-BERT	2DGNN-BERT	BERT
Critical Temperature (1000 epochs)	Small	0.0079	0.0031	0.5627
	Medium	**0.0013**	0.0023	0.3600
Critical Pressure (1000 epochs)	Small	0.0047	0.0017	0.7832
	Medium	**0.0009**	0.0021	0.6278
Critical Volume (1200 epochs)	Small	0.0025	0.0014	0.6480
	Medium	**0.0006**	0.0022	0.4811

Numbers in bold indicate the best results among the three models.

**Table 5:** Evaluation metrics (R^2^, MAE, RMSE, MSE) for three critical properties under three optimal models.

Mode	Molecular Information	Metrics	Temperature	Pressure	Volume
Bert	Smiles	R^2^	0.5685	0.6036	0.0541
		MAE	0.0515	0.0471	0.0573
		RMSE	0.1020	0.0933	0.1253
		MSE	0.0104	0.0087	0.0157
Bert+2D-GNN	Smiles+2D	R^2^	0.8591	0.5330	0.8758
		MAE	0.0365	0.0213	0.0300
		RMSE	0.0592	0.0693	0.0538
		MSE	0.0035	0.0048	0.0029
Bert+3D-GNN	Smiles+2D+3D	R^2^	**0.9188**	**0.7926**	**0.9115**
		MAE	**0.0024**	**0.0820**	**0.0446**
		RMSE	**0.0350**	**0.1104**	0.0673
		MSE	**0.0012**	**0.0122**	0.0045
SVR	2Ddescriptor	R^2^	0.5244	0.3823	0.8856
		MAE	0.0593	0.0779	0.0426
		RMSE	0.0819	0.2031	0.0709
		MSE	0.0067	0.0412	0.0050
SVR+3D	2D+3Ddescriptor	R^2^	0.5870	0.5457	0.7338
		MAE	0.0442	0.0694	0.0793
		RMSE	0.0764	0.1741	0.1082
		MSE	0.0058	0.0303	0.0117
RF	2Ddescriptor	R^2^	0.6333	0.3186	0.4817
		MAE	0.0473	0.0756	0.1093
		RMSE	0.0714	0.2133	0.1510
		MSE	0.0051	0.0455	0.0228
RF+3D	2D+3Ddescriptor	R^2^	0.8381	0.3160	0.8745
		MAE	0.0209	0.0759	0.0407
		RMSE	0.0480	0.2137	**0.0529**
		MSE	0.0023	0.0456	**0.0028**

Numbers in bold indicate the best results among the three models.

**Table 6:** Validation Results from Stratified Independent Test Sets.

Mode	Pre-Training Metrics	Metrics	Temperature
Bert+3D-GNN	Accuracy	R^2^	0.8951
	0.9688	MAE	0.059
	Loss	RMSE	0.0750
	0.003216	MSE	0.0056

**Table 7:** Evaluation Metrics for Baseline Methods.

Mode	Metrics	Temperature	Pressure	Volume
Bert+3D-GNN	R^2^	0.9043	0.7566	0.8905
	MAE	30.171 K	0.604 MPa	29.924 cm^3^/mol
	RMSE	41.494 K	0.813 Mpa	47.007 cm^3^/mol
	MSE	1721.765 K^2^	0.661 MPa^2^	2209.680 (cm^3^/mol)^2^
Chemprop	R^2^	0.560	0.7312	0.867
	MAE	53.02 K	0.6186 MPa	47.45 cm^3^/mol
	RMSE	91.53 K	0.9915 MPa	75.07 cm^3^/mol
	MSE	8377.74 K^2^	0.9846 MPa^2^	2994.28 (cm^3^/mol)^2^
SchNet	R^2^	0.484	0.6281	0.767
	MAE	53.93 K	0.5336 MPa	47.45 cm^3^/mol
	RMSE	99.04 K	1.1667 MPa	75.07 cm^3^/mol
	MSE	9808.92 K^2^	1.3620 MPa^2^	5635.5 (cm^3^/mol)^2^
Joback–Reid	R^2^	0.763	0.632	0.883
	MAE	36.530 K	0.412 MPa	16.267 cm^3^/mol
	RMSE	65.490 K	0.960 MPa	49.21 cm^3^/mol
	MSE	4288.94 K^2^	0.9216 MPa^2^	2421.62 (cm^3^/mol)^2^

## Data Availability

The data repository, including all Supplementary Materials, is publicly accessible at https://github.com/bangeyuelian/GNN-BERT-critical-properties, accessed on 13 September 2026.

## Supplementary Materials

The following supporting information can be downloaded at: https://www.mdpi.com/article/10.3390/molecules31183251/s1, Table S1: Pre-training accuracy and loss of critical-temperature under different training epochs; Table S2: R^2^, MAE, and MSE of the critical temperature prediction task across different epochs; Table S3: Pre-training accuracy and loss of critical pressure under different training epochs; Table S4: R^2^, MAE, and MSE for the critical pressure prediction task across different epochs; Table S5: Pre-training accuracy and loss of critical volume under different training epochs.

## Abbreviations

The following abbreviations are used in this manuscript:

BERT	Bidirectional Encoder Representations from Transformers
QSPR	Quantitative Structure–Property Relationship
SMILES	Simplified Molecular Input Line Entry System
GNN	Graph Neural Networks
SVR	Support Vector Regression
RF	Random Forest
