# Supplementary material for: Integrating Molecular Semantics and Three-Dimensional Geometry for Critical Property Prediction: A Multimodal GNN-BERT Framework
Source: Molecules. 2026 Sep 14;31(18):3251. doi: 10.3390/molecules31183251 (PMC13609510; doi:10.3390/molecules31183251)
Supplement: Supplementary file 1 [file molecules-31-03251-s001.zip › molecules-4529149-supplementary.pdf]

Table S1. Pre-training accuracy and loss of critical-temperature under different training epochs.

	Mode	Metrics/Epoch	400	800	1000	1200	1500	2000
3DGNN-BERT	Small	Accuracy		0.9339	0.9143	0.9542	0.9385	0.93
		Loss		0.0156	0.0079	0.0124	0.0079	
	Medium	Accuracy	0.9323	0.9770	0.9877	0.9786	0.9769	
		Loss	0.0114	0.0025	0.0013	0.0032	0.0049	
	Large	Accuracy	0.9496	0.9781	0.9729			
		Loss	0.0075	0.0033	0.0038			
2DGNN-BERT	Small	Accuracy		0.9239	0.9280	0.9406		
		Loss		0.0031	0.0031	0.0013		
	Medium	Accuracy		0.9262	0.9385	0.9380		
		Loss		0.0030	0.0023	0.0015		
	Large	Accuracy	0.9075	0.9260				
		Loss	0.0045	0.0041				
BERT	Small	Accuracy		0.7845	0.8014	0.7672		
		Loss	0.7728	0.6268	0.5627	0.7923		
	Medium	Accuracy			0.8190	0.9138	0.8707	
		Loss			0.5273	0.3600	0.3567	
	Large	Accuracy	0.8966	0.9052	0.8362			
		Loss	0.4054	0.3058	0.8624			

Table S2. R^2, MAE, and MSE of the critical-temperature prediction task across different epochs.

	Mode		400	800	1000	1200	1500
3DGNN-BERT	Small	R2		0.4852		0.7124	0.4590
		MAE		0.0226		0.0151	0.0152
		MSE		0.0012		0.0007	0.0010
	Medium	R2		0.4737	0.8777	0.8377	0.3672
		MSE		0.0022	0.0137	0.0123	0.0242
		MAE		0.0012	0.0007	0.0004	0.0011
	Large	R2	0.6347	0.2780			
		MAE	0.0176	0.0315			
		MSE	0.0008	0.0020			
2DGNN-BERT	Small	R2		0.8526	0.8462	0.8196	
		MAE		0.0115	0.0117	0.0108	
		MSE		0.0004	0.0004	0.0005	
	Medium	R2	0.8307		0.8591		
		MSE	0.0560		0.0365		
		MAE	0.0085		0.0035		
	Large	R2		0.7101			
		MAE		0.0187			
		MSE		0.0007			
BERT	Small	R2	0.4677				
		MAE	0.0331				
		MSE	0.0063				
	Medium	R2			0.5685		0.1085
		MSE			0.0515		0.0515
		MAE			0.0104		0.0104
	Large	R2		0.1867			
		MAE		0.0542			
		MSE		0.0096			

Table S3. Pre-training accuracy and loss of critical pressure under different training epochs.

	Mode	Metric	400	600	800	1000	1200	1500
3DGNN-BERT	Small	Accura		0.9735	0.9926	0.9716	0.9720	
		Loss		0.0044	0.0023	0.0047	0.0050	
	Medium	Accura			0.9814	0.9931	0.9868	0.9916
		Loss			0.0010	0.0009	0.0022	0.0006
	Large	Accura	0.9655		0.9795			
		Loss	0.0084		0.0044			
2DGNN-BERT	Small	Accura			0.9318	0.9332	0.9165	0.9318
		Loss			0.0028	0.0017	0.0026	0.0021
	Medium	Accura			0.9144	0.9225	0.9222	0.9144
		Loss			0.011	0.0021	0.0010	0.0011
	Large	Accura	0.8712		0.9132			
		Loss	0.0042		0.0022			
BERT	Small	Accura			0.6568	0.6509	0.7396	
		Loss			0.7809	0.7832	0.7631	
	Medium	Accura			0.8166	0.8461	0.8580	
		Loss			0.6960	0.6278	0.5258	
	Large	Accura	0.8284		0.8327			
		Loss	0.5908		0.5742			

Table S4. R^2, MAE, and MSE for the critical-pressure prediction task across different epochs.

	Mode		400	600	800	1000	1200	1500
3DGNN-BERT	Small	R2		0.0633	0.1016		0.2721	
3DGNN-BERT	Small	MAE		0.0185	0.0193		0.0160	
3DGNN-BERT	Small	MSE		0.0015	0.0067		0.0005	
3DGNN-BERT	Medium	R2			0.5237	0.6556	0.6533	
3DGNN-BERT	Medium	MAE			0.0144	0.0167	0.0113	
3DGNN-BERT	Medium	MSE			0.072	0.0037	0.0042	
3DGNN-BERT	Large	R2			0.5662			
3DGNN-BERT	Large	MAE			0.0113			
3DGNN-BERT	Large	MSE			0.0003			
2DGNN-BERT	Small	R2			0.4071	0.4830	0.4209	
2DGNN-BERT	Small	MAE			0.0311	0.0282	0.0214	
2DGNN-BERT	Small	MSE			0.0084	0.0063	0.0074	
2DGNN-BERT	Medium	R2			0.4850	0.5330	0.5014	
2DGNN-BERT	Medium	MAE			0.0239	0.0213	0.0185	
2DGNN-BERT	Medium	MSE			0.0097	0.0048	0.0083	
2DGNN-BERT	Large	R2	0.3480		0.6009			
2DGNN-BERT	Large	MAE	0.0108		0.0112			
2DGNN-BERT	Large	MSE	0.0004		0.0002			
BERT	Small	R2						
BERT	Small	MAE						
BERT	Small	MSE						
BERT	Medium	R2				0.6036		
BERT	Medium	MAE				0.0471		
BERT	Medium	MSE				0.0087		
BERT	Large	R2						
BERT	Large	MAE						
BERT	Large	MSE						

Table S5. Pre-training accuracy and loss of critical volume under different training epochs

	Mode	Metrics/	400	800	1000	1200	1500
3DGNN-BERT	Small	Accurac			0.9843	0.9870	0.9867
		Loss			0.0016	0.0025	0.0028
	Medium	Accurac			0.9870	0.9890	0.9867
		Loss			0.0028	0.0006	0.0028
	Large	Accurac	0.9759	0.9412			
		Loss	0.0084	0.0232			
2DGNN-BERT	Small	Accurac			0.8758	0.9248	0.9154
		Loss			0.0118	0.0014	0.0037
	Medium	Accurac			0.9005	0.9157	0.9126
		Loss			0.0079	0.0022	0.0011
	Large	Accurac	0.8712	0.9132			
		Loss	0.0042	0.0022			
BERT	Small	Accurac			0.7455	0.7768	0.8027
		Loss			0.7296	0.6480	0.6271
	Medium	Accurac			0.8205	0.8561	0.8462
		Loss			0.5867	0.4811	0.5263
	Large	Accurac	0.8205	0.7949			
		Loss	0.6013	0.637			
